# Pediatric Deep Venous Thrombosis and Pulmonary Embolism: Can It Be Antiphospholipid Syndrome?

**DOI:** 10.4274/tjh.galenos.2018.2018.0214

**Published:** 2019-08-02

**Authors:** Fatma Demir Yenigürbüz, Hale Ören

**Affiliations:** 1 Dokuz Eylül University Faculty of Medicine, Department of Pediatric Hematology, İzmir, Turkey

**Keywords:** Deep venous thrombosis, Pulmonary embolism, Antiphospholipid syndrome

## To the Editor,

**Figure 1:**
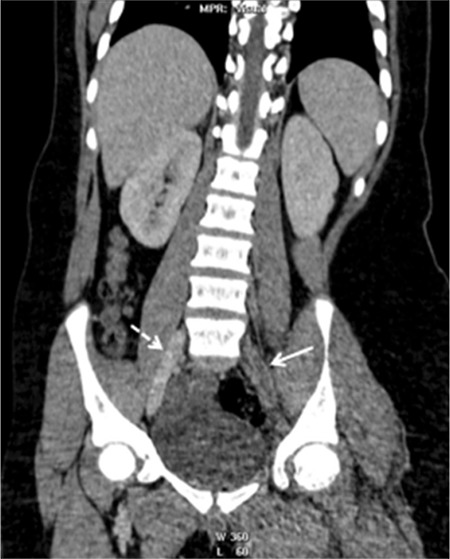
Abdominal computed tomography of the patient demonstrating iliac vein thrombosis (arrows).

**Figure 2:**
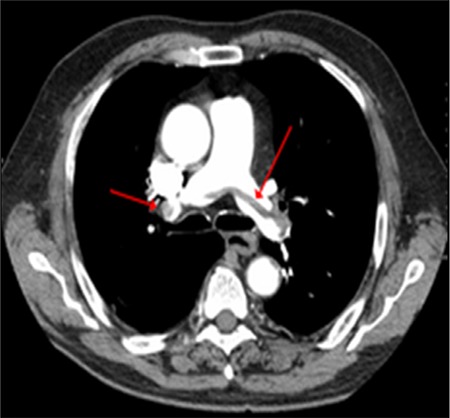
Thorax computed tomography angiography of the patient demonstrating filling defects in right pulmonary artery (arrows).

In pediatric patients with deep venous thrombosis (DVT) and pulmonary embolism (PE), antiphospholipid syndrome (APS) should be considered early and efforts must be made to ensure timely diagnosis of this potentially life-threatening condition. Pediatric APS is an autoimmune disease characterized by vascular thrombosis and persistently positive antiphospholipid antibodies [1,2,3,4,5]. Primary APS is rarely seen in childhood [4]. A 14-year-old adolescent was admitted with complaints of left upper leg edema for 1 week. On physical examination, obesity, hypertension, and edema of the leg were present. Hyperlipidemia and D-dimer elevation were remarkable. Doppler ultrasonography showed DVT in his left femoral vein and abdominal computed tomography (CT) demonstrated iliac vein thrombosis (Figure 1). Since he had widespread DVT, thorax CT angiography was also performed without any clinical symptoms of PE and it demonstrated filling defects in the right pulmonary artery (Figure 2). Anticoagulation was given and complete recanalization was observed. A diet program was started. When thrombophilia risk factors were evaluated, there was no family history and the genetic thrombophilia panel was negative, LA was positive twice with an interval of 12 weeks (first sample was before treatment), and other APS antibodies were found negative. Systemic lupus erythematosus (SLE) and SLE-like diseases were excluded. The patient was diagnosed with primary APS. Metabolic syndrome was the additional thrombotic risk factor. Long-term anticoagulation therapy (lifetime) was given to the patient.
